# Decoupling between Economic Development and Carbon Emissions and Its Driving Factors: Evidence from China

**DOI:** 10.3390/ijerph19052893

**Published:** 2022-03-02

**Authors:** Xiaochun Zhao, Mei Jiang, Wei Zhang

**Affiliations:** 1 School of Management, Anhui University, Hefei 230601, China; 14006@ahu.edu.cn (X.Z.); l21201016@stu.ahu.edu.cn (M.J.); 2 School of Public Administration, Sichuan University, Chengdu 610065, China

**Keywords:** carbon emissions, Tapio decoupling, LMDI model, provincial level, low-carbon economy

## Abstract

Analyzing the relationship between economic development and carbon emissions is conducive to better energy saving and emission reduction. This study is based on the panel data of China’s carbon emissions, from 2009 to 2019, and quantitative analysis of the relationship between carbon emissions and economic development through the Tapio decoupling model and the Logarithmic Mean Divisia Index (LMDI) decomposition model. The results show that: First, carbon emission and economic development are increasing year by year, and the development trend of economic growth rate and carbon emission growth rate presents the characteristics of consistency and stage. Second, China’s carbon emissions and economic development are basically in a weak decoupling state, and carbon emissions and economic development are positively correlated. Third, there are significant differences in decoupling indices among the four regions, mainly in that the central region is better than the eastern region, the eastern region is better than the northeast region, the northeast region is better than the western region, and the development of provinces in the region is unbalanced. Fourth, from the perspective of driving factors, the elasticity of population size and economic intensity can restrain the decoupling of carbon emissions, while the elasticity of energy intensity and carbon intensity have a positive effect. Finally, according to the results of empirical analysis, this paper focuses on promoting China’s emission reduction and energy sustainable development from the aspects of developing low-carbon and zero carbon technology, supporting new energy industries and promoting the construction of a carbon emission trading market.

## 1. Introduction

In recent years, global warming has led to increasingly frequent extreme weather, which seriously threatens human survival and development. For example, in July 2021, the extreme rainstorm in Henan Province caused 302 people to lose their lives and a direct economic loss of CNY 53.2 billion. Therefore, global warming caused by carbon dioxide emissions has become a topic of increasing concern [1]. As early as the First World Climate Conference held in Geneva in 1979, the issue of global climate warming was raised for the first time. People realized that the global climate change caused by carbon emissions in the industrial era has already threatened human development; thus, measures must be taken to reduce carbon emissions. At present, as the world’s largest energy consumer [2], China ranks first in the world in terms of carbon emissions and faces tremendous pressure to reduce emissions. At the same time, as an active participant in emission reduction, the development of a low-carbon economy has always been of great concern for the Chinese government. Based on the basic national conditions and the status quo of social development, the Chinese government has been active in promoting its carbon neutral action plan, and has made certain achievements in reducing the intensity of carbon emissions [3], but the carbon emission growth rate remains fast as a result of rapid economic growth. From 2009 to 2019, China’s gross domestic product (GDP) increased by 2.6 times, and its carbon emissions increased by 1.5 times. In order to increase emission reduction efforts, the Chinese government announced that it would realize the peak carbon dioxide emissions by 2030 and carbon neutrality by 2060. Since carbon dioxide emissions are mainly produced in the process of economic development, this paper believes that we should first clarify the relationship between economic development and carbon emissions in order to achieve carbon neutralization more efficiently. Only by clarifying the relationship between them can we take targeted policy to achieve carbon neutrality at a faster speed.

Therefore, this study takes China as an example, which is divided into four regions: eastern, northeast, central and western. From the perspective of decoupling analysis, it makes an empirical analysis of the relationship between economic development and carbon emissions in four regions of China. Combined with the LMDI driving factor decomposition model, it studies which factors play the main role in decoupling carbon emissions, and on this basis, it takes targeted measures to provide intellectual support for further promotion of the sustainable development of China’s green economy. As the largest developing country in the world, the analysis of the relationship between China’s economic development and carbon emissions can provide some inspiration for other countries in the world to better reduce carbon emissions.

The arrangement of this paper is as follows: It first sorts out the related research results of economic development and carbon emissions. Afterwards, it introduces the research methods and data sources of this paper. Next, it displays and discusses the specific research results. Finally, it mainly puts forward the research findings and corresponding policy recommendations and summarizes the shortcomings of this paper.

## 2. Literature Review

### 2.1. Relationship between Carbon Emission and Economic Development

Scholars have performed considerable research on the relationship between carbon emissions and economic development [4]. According to the literature review, it was found that the existing literature mainly focuses on the application of research methods and commonly uses research methods including the Granger causality test analysis, EKC hypothesis and decoupling model. First, Granger causality is used to analyze the correlation between two variables; it can play a certain role in economic forecasting. For example, Kofi et al. (2017) found that there was a long-term one-way causal relationship between energy consumption and economic growth in China using the Granger causality analysis and proposed that China should transform its trade growth mode [5]. Melike et al. (2019) studied Granger causality between carbon dioxide emissions and economic growth in China and the United States and found that there was a one-way causal relationship between carbon emissions and economic growth in China, but it has not been the case in the United States. The results showed that different regions should adopt different policy recommendations [6]. Second, by analyzing the relationship between economic development and carbon emissions, scholars use the Environmental Kuznets Curve (EKC) as an analytical perspective [7]. EKC reveals that there is an inverted U-shaped relationship between the environment and the economy, which is a powerful tool for studying environmental problems and economic development. For example, Abdul et al. (2018) selected BRICS countries as research objects and analyzed the relationship between financial development, globalization, economic growth, energy consumption, urbanization and carbon emissions of BRICS countries based on the existence of the EKC hypothesis and supported the EKC hypothesis in BRICS countries [8]. Subsequently, the academic circle has conducted many studies on whether the hypothesis of an inverted U-shaped curve is correct. Awaworyi et al. (2018) analyzed the curves of the relationship between environmental pollution and economic development of 20 Organization for Economic Cooperation and Development (OECD) countries through the panel data estimator, and found that only nine countries were in line with the EKC hypothesis. Moreover, there appeared multiple shapes of the relationship curve such as an inverted U, an inverted N and N-shape, but no single shape was applicable to all regions [9]. Pan et al. (2017), based on the data of India and China from 1971 to 2012, discussed the cointegration relationship among carbon emissions and economic activities and tested the EKC hypothesis, thus discovering that the relationship between economic growth and carbon emissions was N-shaped, which deviated from the EKC hypothesis [10]. Finally, in order to clarify the state between economic development and carbon emissions in different regions at different times, academic circles generally use decoupling model for empirical analysis [11]. To analyze the relationship between the European Union (EU) and Finland road traffic flow, carbon dioxide emissions, and GDP from 1970 to 2001, Petri Tapio (2005) introduced decoupling elasticity into decoupling research for the first time, and further subdivided the concepts of decoupling, coupling and negative decoupling, so as to set eight decoupling indicators according to the ratio of carbon emission change to economic development change. The study showed that the relationship between carbon dioxide produced by transportation and GDP was weakly decoupled [12]. Karmellos et al. (2021), based on decoupling theory, analyzed the decoupling state between carbon emissions generated by electricity and economic growth in the EU and discovered that most countries were in a strong decoupling state from 2013 to 2018 [13]. Smbi et al. (2021) used the gravity model, LMDI model and Tapio decoupling model to study the decoupling state between economic development and carbon emissions in Africa, and the results showed that many countries in Africa showed obvious negative decoupling and weak decoupling [14]. Xin et al. (2021) used the two-dimensional decoupling model to explore the dynamic decoupling relationship between economic development and carbon emissions in Gansu Province from 2000 to 2017. The results showed that the two-dimensional decoupling state of Gansu Province was low-level weak decoupling [15].

### 2.2. Decomposition of Carbon Emission Drivers

In terms of analyzing the decomposition of carbon emission driving factors, scholars have carried out many studies, and the current research methods include the structural decomposition model and exponential decomposition model [16]. The structural decomposition model is used to analyze the change in dependent variables by the change of independent variables. Wang et al. (2017) decomposed the carbon emissions of Guangdong Province based on the structural decomposition model and found that economic and population growth had positive effects on carbon emissions, and the intensity of carbon emissions was the main factor in restraining carbon emissions [17]. Vries et al. (2017) used the structural decomposition model to analyze the decoupling between carbon emissions and economic growth in developed and emerging economies, and decomposed the driving factors into global supply chain participation, consumption and technology [18]. Ninpanit et al. (2019) carried out the structural decomposition analysis (SDA) to study the factors that led to the change in carbon emissions in Thailand, and found that the increase in per capita consumption in Thailand and abroad had obvious influences on the growth of carbon emissions, but the improvement of energy efficiency was not enough to reduce emissions [19].The exponential decomposition model is a method of decomposing the change of the independent variable into the change of the dependent variable [20]. Román et al. (2018) constructed the Index Decomposition Analysis–Logarithmic Mean Divisia Index (IDA-LMDI) model to analyze the driving factors of carbon emissions in Colombia, and pointed out that income and population are the main driving factors affecting carbon emissions [21]. Yang et al. (2021) used the exponential decomposition model to analyze the driving factors of carbon emissions based on the carbon emission panel data of 78 regions and found that the production efficiency and energy-saving technology had positive effects on reducing global carbon emissions, while the growth of per capita GDP and population growth had inhibitory effects on global carbon emissions [22]. Liu et al. (2021) proposed that seven factors had driving effects on carbon emissions based on the decoupling relationship between carbon emissions and economic growth in China’s transportation industry [23], and technical effects were the main factors in restraining carbon emissions. To sum up, scholars have conducted in-depth discussions on the research of carbon emission drivers, but most of them are confined to a certain sub-sector, such as transportation, non-ferrous metal industry, and the agriculture and industrial sectors, or are limited to a certain province or region. Little research has been performed on China’s overall carbon emission drivers, and to ensure that the results are reliable and have no residuals [24], this paper mainly uses the LMDI model to analyze China’s carbon emission drivers.

The research results of these studies are beneficial to reduce carbon emissions, but they still have shortcomings. First, most scholars’ analyses of decoupling between carbon emissions and economic development is limited to grade assessment, without analyzing the deep causes of decoupling. Second, the literature analyzing the driving factors of carbon emissions focuses only on a certain industry or region, and there is a lack of research on the driving factors of carbon emissions in China as a whole. Therefore, in order to fill the above research gap, based on the carbon emission panel data of 30 provinces in China, this paper decided to study the decoupling status of economic development and carbon emissions using the Tapio decoupling model and constructs of the LMDI model to explore the driving factors that affect the decoupling of carbon emission, so as to analyze the underlying reasons of China’s current decoupling state, with a view to providing policy inspirations for China to realize peak carbon dioxide emissions and carbon neutrality.

## 3. Research Methods and Data Sources

### 3.1. Research Methods

#### 3.1.1. Carbon Emission Measurement

Since the China Energy Statistics Yearbook does not have official data on carbon emissions of Chinese provinces, this study refers to the carbon emission coefficient method of energy consumption described by the United Nations Intergovernmental Panel on Climate Change (IPCC) and draws on the research results of Fan et al. (2019) to choose eight carbon sources such as coal, coke, kerosene and natural gas to calculate carbon emissions in combination with the carbon emission coefficient of each energy source [25]. The specific formula is as follows:

(1) $$C=\overset{8}{\underset{j=1}{\sum }}E_{j}\times \alpha_{j}\times \xi_{j}$$

where *C* represents carbon emission, *E_j_* represents the physical consumption of the *j*-th energy, *α_j_* represents the coefficient of standard coal, and *ξ_j_* represents the carbon emission coefficient.

#### 3.1.2. Tapio Decoupling Model

**Figure 1:**
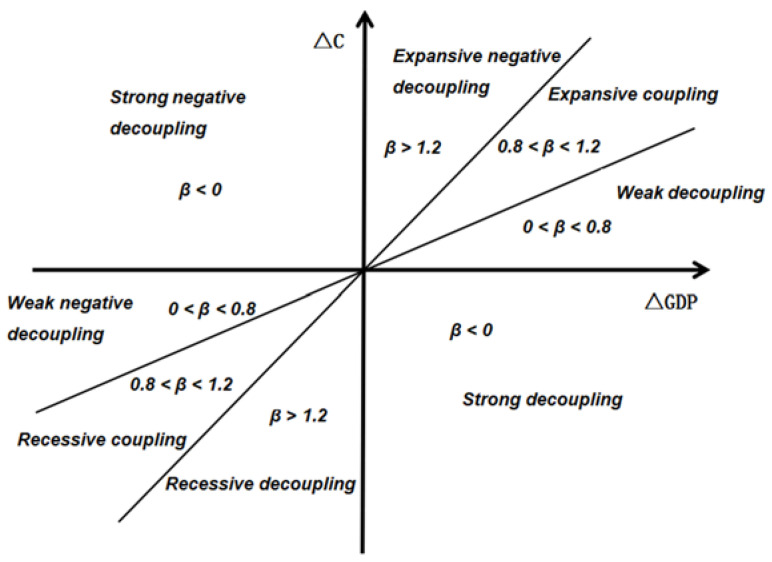
Decoupling state division.

“Decoupling” refers to the trend that the relationship between economic development and environmental pollution is constantly separated. This concept was gradually extended from agricultural policy research to environmental field by OECD. In this study, “decoupling” is used to describe the gradual reduction of carbon emissions with economic growth. Currently, widely used decoupling models mainly include the Organization for Economic Co-operation and Development (OECD) decoupling index method and Tapio decoupling index method. Compared with the OECD decoupling model, the Tapio decoupling index method can better reflect the decoupling state between economic development and carbon emissions without considering the limitation of base period [26]. Therefore, this study uses the Tapio decoupling model to measure China’s decoupling elasticity, and the specific formula is as follows:

(2) $$\beta =\frac{\Delta C/C}{\Delta GDP/\mathrm{GDP}}$$

where *β* represents the decoupling index, *C* and GDP represent the base period values of carbon emissions and GDP, respectively, while △*C* and △*GDP* represent the difference between current period and base period of carbon emissions and GDP. The Tapio decoupling model is specifically divided into eight decoupling states, as shown in Figure 1.

#### 3.1.3. LMDI Driving Factor Decomposition Model of Carbon Emission

The factor decomposition method includes the structure decomposition method and exponential decomposition method. The exponential decomposition method has the advantages of no residual error and strong applicability and can enhance the reliability of the results [27]; thus, the LMDI exponential decomposition method is adopted in this paper. Drawing on the basic principle of Kaya identity, the formula for carbon emission can be expressed as:

(3) $$C=P\times \frac{G}{P}\times \frac{E}{G}\times \frac{C}{E}=p\times g\times e\times s$$

where *P* represents the population, *G* represents the gross domestic product, *E* represents the energy consumption, *C* represents the carbon emissions, *p* represents population size, *g* represents per capita GDP, *e* represents energy intensity and *s* represents carbon emission intensity. According to LMDI addition decomposition, the carbon emission change Δ*C* in the target year relative to the base year can be decomposed into:

(4) $$\Delta C=\Delta C^{t}-\Delta C^{o}=\Delta C_{p}+\Delta C_{g}+\Delta C_{e}+\Delta C_{s}$$

where △*C_p_* represents population effect, △*C_g_* represents economic intensity effect, △*C_e_* represents energy intensity effect and △*C_s_* represents carbon intensity effect. Among them:

(5) $$\begin{array}{l} \Delta C_{p}=\sum W\times \ln\frac{C_{p}^{t}}{C_{p}^{o}}\\ \Delta C_{g}=\sum W\times \ln\frac{C_{g}^{t}}{C_{g}^{o}}\\ \Delta C_{e}=\sum W\times \ln\frac{C_{e}^{t}}{C_{e}^{o}}\\ \Delta C_{s}=\sum W\times \ln\frac{C_{s}^{t}}{C_{s}^{o}}\\ W=\frac{\left(C^{t}-C^{o}\right)}{\ln(C^{t}/C^{o})} \end{array}$$

By combining Equations (2) and (4), it can be concluded that:

(6) $$\begin{array}{l} \beta =\Delta C\times \frac{GDP}{C\times \Delta GDP}=(\Delta C_{p}+\Delta C_{g}+\Delta C_{e}+\Delta C_{s})\times \frac{GDP}{C\times \Delta GDP}=\frac{\Delta C_{p}\times GDP}{C\times \Delta GDP}\\ +\frac{\Delta C_{g}\times GDP}{C\times \Delta GDP}+\frac{\Delta C_{e}\times GDP}{C\times \Delta GDP}+\frac{\Delta C_{s}\times GDP}{C\times \Delta GDP}=f_{p}+f_{g}+f_{e}+f_{s} \end{array}$$

where *f_p_* represents the decoupling index of population, *f_g_* represents the decoupling index of economic intensity, *f_e_* represents the decoupling index of energy intensity and *f_s_* represents the decoupling index of carbon intensity.

### 3.2. Data Sources

This paper selects the national data from 2009 to 2019 for the empirical analysis. Economic growth is expressed by gross domestic product (GDP). GDP data and population data come from the China Statistical Yearbook (2010–2020), and energy consumption data come from the China Energy Statistical Yearbook (2010–2020). There are eight types of energies in statistics, which are uniformly converted into ten thousand tons of standard coal. Based on the availability of data, this paper takes 30 provinces, cities and municipalities in China as the research object, not including Tibet, Hong Kong, Macao and Taiwan.

## 4. Results

### 4.1. Characteristics of Economic Development and Carbon Emissions

**Figure 2:**
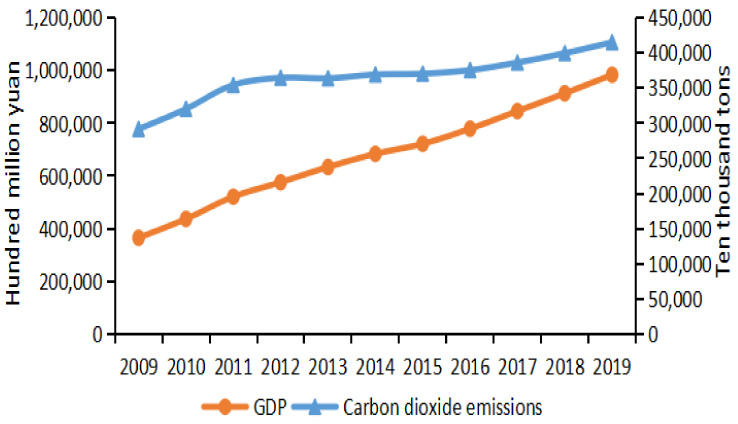
Carbon emissions and economic growth trend (2009–2019).

**Figure 3:**
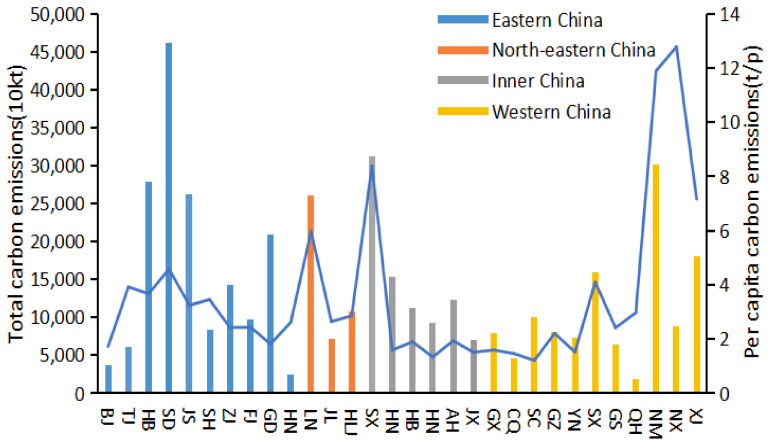
Total carbon emissions and per capita carbon emissions of province.

According to Equation (1), this study can calculate China’s annual carbon emissions from 2009 to 2019. The specific calculation results are shown in Figure 2. In order to better understand the differences in carbon emissions among Chinese provinces, the specific calculation results are shown in Figure 3.

On the whole, from 2009 to 2019, China’s carbon emissions and economic development level increased year by year. This shows that in China, the largest energy consumer, a large amount of energy consumption not only brings rapid economic development, but also leads to an increase in carbon emissions [28]. It can be seen that the level of economic development is closely related to the growth rate of carbon emissions. However, Figure 2 also shows that the growth trend of carbon emission has tended to level off in the past decade under the rapid growth of the economy, showing that China’s low-carbon development has achieved some results in recent years. According to the situation of carbon emissions in Figure 2, it can be divided into two stages: the first stage from 2009 to 2012 and the second stage from 2012 to 2019. In the first stage, carbon emissions increased rapidly, while in the second stage, carbon emissions tended to flatten out. The reason for the rapid growth in the first stage may be that the Chinese government strengthened energy consumption after the world financial crisis in 2008 in order to promote economic recovery. The reason for the slowing of carbon emissions in the second phase is that the eighteenth national congress of the Chinese government in 2012 put forward a strategic decision to vigorously promote the construction of ecological civilization. As the basis of the Overall Plan for Development in Five Areas, the building of an eco-civilization has become the focus of the Chinese government. Following the concept of “lucid waters and lush mountains are invaluable assets”, China has made great improvement in cutting carbon emissions by implementing effective policies [29].

In order to better understand the characteristics of China’s carbon emissions, China’s 30 provinces are divided into eastern, northeastern, central and western regions (see Figure 3). The average carbon emissions of eastern China were higher than the northeast, central and western regions, and the economic development level of the eastern region was also higher than the other three regions, indicating that there is a positive correlation between economic development and carbon emissions. According to the provincial data, Hebei, Shandong and Jiangsu provinces have the highest carbon emissions, and the economic development level of these three provinces ranks the top ten in China. Among these three provinces, Jiangsu’s carbon emissions are relatively low, but the economic development level is the highest. The main reason is that Jiangsu province is committed to improving energy efficiency and developing emerging industries such as information technology to promote economic development [30], while Shandong and Hebei provinces rely on heavy industry to develop their economy [30], and infrastructure construction is imperfect, industrial energy consumption is large, and the introduction rate of energy-saving technology is low. Therefore, it is urgent that the two provinces increase the policy support. Among them, Beijing, Hainan and Qinghai provinces have the lowest carbon emissions. The main reason is that the population sizes in these three provinces are much smaller than that of other regions. Moreover, Beijing ranks first in terms of the economic development level among these three provinces. As the political center of China, Beijing responds quickly to the green and low-carbon policy and has a great advantage in transportation [31]. It has adopted many effective measures to cut emissions and speed up the transformation and upgrading of the economy. From Figure 3, there are differences between the results of per capita carbon emissions and total carbon emissions in each province. The per capita carbon emissions in Liaoning, Shanxi, Inner Mongolia and Ningxia provinces are much higher than those in Hebei, Shandong and Jiangsu provinces, where the total carbon emissions are relatively high. The reason is that the regional carbon emissions are affected by the population [32]. To sum up, China’s carbon emissions not only exist as regional imbalances, but also, the provinces within the region have significant differences due to reasons such as population size and economic development level [33].

**Figure 4:**
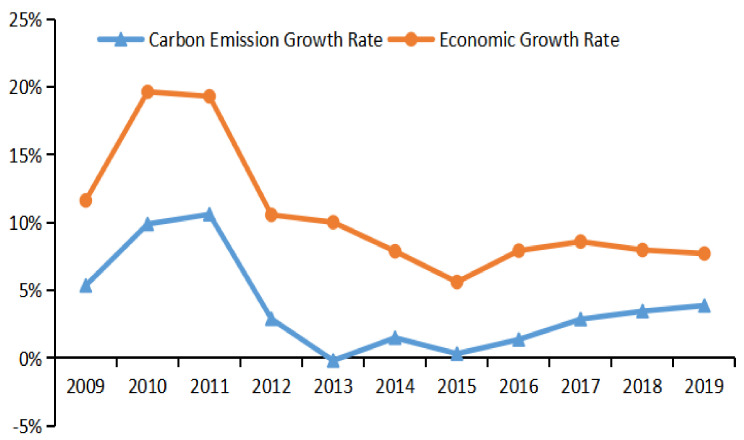
General trend chart of carbon emission growth rate and economic growth rate (2009–2019).

It can be seen from Figure 4 that from 2009 to 2019, China’s economic growth rate was higher than the growth rate of carbon emissions, and the economic growth rate was consistent with the growth rate of the carbon emissions. Since 2012, the growth rate of carbon emissions has been below 5% (see Figure 4). It can be seen that the concept of green and low carbon has played a certain role in restraining carbon emissions, and the Chinese government has also made great efforts to reduce carbon emissions [34]. From 2011 to 2013 and from 2014 to 2015, the growth rate of carbon emissions decreased significantly, showing that the Chinese government complied with the theme of scientific development and the main line of accelerating the transformation of economic development mode in the 12th Five-Year Plan to effectively solve the problems between the economy and environment [35]. The results also show that the vigorous progress made in the 12th Five-Year Plan has become an important turning point on China’s emission reduction road [36], laying a foundation for the slowdown of carbon emissions growth and the improvement of economic development efficiency. 

### 4.2. Decoupling Analysis of China’s Economic Growth and Carbon Emissions

The above section analyzes the overall characteristics of carbon emissions in different regions of China and has a systematic understanding of carbon emissions and economic development in different regions of China. Next, this paper uses the Tapio decoupling model, namely Equation (2), to analyze the decoupling between China’s economic growth and carbon emissions from 2009 to 2019, and the results are shown in Table 1.

Generally speaking, from 2009 to 2019, China’s 30 provinces were basically in a weak decoupling state, and carbon emissions were positively correlated with economic development. During this period, the average growth rate of carbon emissions was 3.6%, while the average growth rate of economic development level was 10.5%. As the economic growth rate was higher than that of carbon emissions, carbon emissions and economic development were in a weak decoupling state, which showed that China, as a big energy country, still has a high dependence on energy consumption to developed economy. According to the fluctuation of the decoupling index, it can be seen that China’s decoupling index gradually decreased from 2009 to 2019, which was mainly due to the active measures taken by the Chinese government to transform industrial structures [34], actively developing a green and low-carbon circular development industrial system under the high attention of the international environment to climate issues.

There are significant differences in China’s decoupling situation (see Table 1), which mainly shows that the central region is better than the eastern region, the eastern region is better than the northeast region, the northeast region is better than the western region, and the decoupling elasticity of carbon emissions and economic development in the western region was always lower than the average decoupling elasticity in China. The six central provinces share similar conditions of economy and population, and their average decoupling index shows little diverseness. However, Shanxi Province has the highest decoupling index, mainly because Shanxi, as an energy-based province and major coal producer, has a slow rate of economic growth and a strong dependence on energy, and is still at the exploration stage of energy transformation [37]. From 2017 to 2019, Henan Province was in a strong decoupling state. The reason is that the economic development level of Henan has been in the forefront of the country and the industrial structure has continuously been optimized, achieving economic growth while improving environmental benefits [38]. However, there are significant differences in the decoupling situation among the eastern provinces, and the high difference in the decoupling index inhibits the average carbon emission index in the eastern region. Among them, Beijing has been in a strong decoupling type at all stages (see Table 1). The decoupling index between Tianjin and Shandong was relatively high, because Shandong Province, as an industrial city, has large demand for energy that has led to carbon emissions. It can be seen that there are significant internal differences in the decoupling elasticity of carbon emissions in the eastern region. Northeast China is rich in coal resources, with heavy industry as the pillar industry causing carbon emissions [39]. Due to the relatively backward economic development in the western region, the application of energy-saving technologies was relatively rare. Ningxia is the most prominent example in this region, and the decoupling state is expansive coupling. The reason for the expansive coupling may be that Ningxia is well positioned in the field of new energy development with its the utilization rate of new energy taking the lead nationwide [40]. The complete infrastructures in the energy industry and the good trend of economic development have caused the rapid increase in carbon emissions.

### 4.3. Decomposition Analysis of Driving Factors of Carbon Emission in China

**Figure 5:**
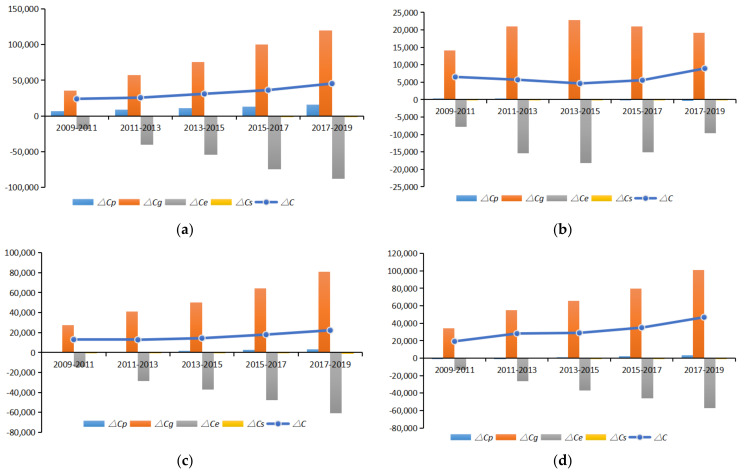
Contribution of various influencing factors of carbon emissions (2009–2019): (**a**) eastern China; (**b**) northeastern China; (**c**) inner China; (**d**) western China.

According to the LMDI-driven decomposition model, namely Equation (3), Equation (4), Equation (5), and Equation (6), the elasticity of carbon emission driving factors in China’s 30 provinces from 2009 to 2019 was measured. It was divided into five stages: 2009–2011, 2011–2013, 2013–2015, 2015–2017, and 2017–2019. The results are shown in Table 2, and the results of the contributions to the four driving factors are shown in Figure 5. 

It can be analyzed from Table 2 that the elasticity of population size and elasticity of economic intensity are basically positive, and these two factors have an inhibition effect on the decoupling state of carbon emissions in various regions of China, and on the whole, the elasticity of economic intensity has the highest value; that is, the elasticity of economic development is the most important inhibition factor for the decoupling of carbon emissions and plays a dominant role in the increase in carbon emissions. It can be seen that various regions of China need to accelerate the transformation of economic development mode. Among the driving factors, energy intensity elasticity and carbon intensity elasticity are negative as a whole, which play major roles in promoting the decoupling of carbon emissions. Among them, carbon intensity elasticity has little influence on the decoupling of carbon emissions, and energy intensity elasticity is the main driving force for restraining carbon emissions. It can be seen that all regions in China need to improve the utilization rate of energy, expand the scope of introducing energy-saving technologies and exert the important influence of energy intensity on the decoupling of carbon emissions. The elasticity of economic intensity of 30 provinces has gradually decreased, and the inhibitory effect on carbon emission decoupling has also decreased. In summary, the Chinese government has made some progress in changing the mode of economic development, which has effectively alleviated the economy’s excessive dependence on energy. Energy intensity elasticity is increasingly important, and the Chinese government also provided great support in improving energy efficiency.

From the contribution of driving factors, the economic intensity of the four regions as a whole contributes greatly to the increase in carbon emissions, while the energy intensity contributes to the reduction of carbon emissions, and the contribution of economic intensity was higher than the contribution of incremental carbon emissions. The contribution has risen year by year, which shows that the economy still has a strong dependence on energy. As it is shown in Figure 5, the population size in the eastern region also plays a role in promoting carbon emissions, while the population size in the other three places has no significant influence. The reason may be that the eastern region has a high level of economic development and large population mobility, forming in a certain population size [41]. The increase in carbon emissions in northeast China shows a trend of first rising and then falling and generally shows a slow upward trend, while an increase in carbon emissions in the other three regions shows an increasing trend year by year. Comparing the contribution values of the four regions’ economic intensity, the research can find that the eastern region is better than the central region, central region is better than the western region, and the western region is better than the northeast region. To sum up, the energy intensity factors can promote the emission reduction, and there are significant regional differences in the contribution values of the influencing factors of carbon emissions in the four regions.

## 5. Conclusions and Discussion

This paper estimates the carbon emissions of 30 provinces in China from 2009 to 2019, applying the Tapio decoupling model to analyze the decoupling state of economic development and carbon emissions and the LDMI model to discuss the driving factors behind carbon emissions. Based on China’s provincial panel data, we can draw the following conclusions: First, from the perspective of the characteristics of economic development and carbon emissions, both carbon emissions and GDP are increasing year by year, and the economic growth rate is relatively fast and the growth trend of carbon emissions is relatively flat, which is consistent with the policy goal of low-carbon economic development. Second, China’s carbon emissions and economic development are basically in a weak decoupling state, and the decoupling index shows a downward trend, from 0.49 to 0.293, which indicates that China’s low-carbon economy has achieved certain results, but overall, there is still much room for improvement in China’s emission reduction. Third, there are significant differences in the decoupling state between carbon emissions and economic development in different regions of China, and the development of provinces in the region is unbalanced. It mainly shows that the central region is better than the eastern region, the eastern region is better than the northeast region, and the northeast region is better than the western region, and only Beijing Province and Henan Province among the 30 provinces and cities have entered a strong decoupling state. Fourth, the elasticity of economic intensity and the elasticity of population size inhibit the decoupling of China’s carbon emissions, while the elasticity of energy intensity and the elasticity of carbon intensity promote it. At the same time, the role of energy intensity in inhibiting carbon emissions is significantly enhanced. Wang and others also found that energy intensity is an important factor in promoting the decoupling process [42], which indicates that China needs to make efforts to improve energy efficiency.

Based on the above research conclusions, in order to promote the construction of China’s low-carbon economic development system, the following suggestions are put forward:

(1) Accelerate the development of energy-saving technologies and improve energy utilization [43]. From the panel data of 30 provinces in China, it is concluded that the energy intensity has a positive effect on the decoupling of carbon emissions. Therefore, the Chinese government should pay attention to the application of green technologies to improve energy efficiency [44].
(2) Adjust measures to local conditions and strengthen regional cooperation. In actively exploring the new path of low-carbon development, we should promote the economically developed areas to reach peak carbon dioxide emissions first, and at the same time strengthen regional cooperation [45]. The economically underdeveloped areas should learn from the experience of carbon reduction and promote the sustainable development of energy.
(3) Promote the optimization and upgrading of industrial structure and support the new energy industry. China focuses on the secondary industry, and it is more feasible to develop clean energy than to reduce coal consumption. In recent years, new energy sources such as solar energy and wind energy have been widely used. The Chinese government can optimize the industrial structure by expanding the proportion of clean energy [46].
(4) Promote the building of the carbon emission trading market. Carbon emission trading market is a powerful tool to reduce carbon emissions by market mechanism and a platform for green and low-carbon economic development. The trading market can effectively reduce the carbon-emission cost, establish a voluntary emission reduction mechanism, and actively promote the regional carbon emission reduction responsibility. Conversely, it is necessary to strengthen the institutional construction of the carbon emission trading market, improve the legal support for the trading market, ensure the standardized operation of the trading market, and promote the strong decoupling between China’s economic development and carbon emissions.

The conclusions drawn from the panel data of China’s economic development and carbon emissions from 2009 to 2019 can provide a reference for China’s low-carbon economic development, but there are still some shortcomings that need to be further improved. First, this study is based on the panel data of 30 provinces only and pays little attention to the characteristics of carbon emissions in different regions within each province; therefore it is limited by the lack of more detailed data, which provide a direction for future research. Second, the driving factors of carbon emissions are diverse and complex, and only four factors are considered in this study. In the future, the research should be further deepened, and other important factors, such as industrial structure and economic structure, should be selected to further improve the research.

## Figures and Tables

**Table 1:** Decoupling between carbon emissions and economic growth.

Area	Province	2009–2011		2011–2013		2013–2015		2015–2017		2017–2019	
		*β*	Decoupling	*β*	Decoupling	*β*	Decoupling	*β*	Decoupling	*β*	Decoupling
Eastern China	Beijing	−0.157	Strong	−0.193	Strong	−0.105	Strong	−0.114	Strong	−0.064	Strong
	Tianjin	0.731	Weak	0.459	Weak	0.295	Weak	0.198	Weak	0.407	Weak
	Hebei	0.513	Weak	0.36	Weak	0.231	Weak	0.149	Weak	0.264	Weak
	Shandong	0.481	Weak	0.307	Weak	0.425	Weak	0.417	Weak	0.532	Weak
	Jiangsu	0.666	Weak	0.471	Weak	0.38	Weak	0.297	Weak	0.248	Weak
	Shanghai	0.466	Weak	0.398	Weak	0.166	Weak	0.128	Weak	0.081	Weak
	Zhejiang	0.349	Weak	0.177	Weak	0.137	Weak	0.135	Weak	0.112	Weak
	Fujian	0.639	Weak	0.304	Weak	0.355	Weak	0.241	Weak	0.257	Weak
	Guangdong	0.594	Weak	0.314	Weak	0.231	Weak	0.231	Weak	0.19	Weak
	Hainan	0.54	Weak	0.231	Weak	0.395	Weak	0.239	Weak	0.235	Weak
	Mean	0.482	Weak	0.283	Weak	0.251	Weak	0.192	Weak	0.226	Weak
Northeast China	Liaoning	0.369	Weak	0.203	Weak	0.147	Weak	0.324	Weak	0.6	Weak
	Jilin	0.609	Weak	0.264	Weak	0.13	Weak	0.095	Weak	0.125	Weak
	Heilongjiang	0.338	Weak	0.199	Weak	0.192	Weak	0.201	Weak	0.224	Weak
	Mean	0.439	Weak	0.222	Weak	0.157	Weak	0.207	Weak	0.317	Weak
Central China	Shanxi	0.349	Weak	0.371	Weak	0.392	Weak	0.421	Weak	0.573	Weak
	Henan	0.502	Weak	0.157	Weak	0.127	Weak	0.04	Weak	−0.032	Strong
	Hubei	0.587	Weak	0.143	Weak	0.106	Weak	0.097	Weak	0.089	Weak
	Hunan	0.369	Weak	0.152	Weak	0.13	Weak	0.136	Weak	0.074	Weak
	Anhui	0.264	Weak	0.311	Weak	0.279	Weak	0.224	Weak	0.165	Weak
	Jiangxi	0.525	Weak	0.429	Weak	0.394	Weak	0.325	Weak	0.279	Weak
	Mean	0.433	Weak	0.26	Weak	0.238	Weak	0.207	Weak	0.191	Weak
Western China	Guangxi	1.058	Expansive	0.791	Weak	0.517	Weak	0.562	Weak	0.545	Weak
	Chongqing	0.482	Weak	0.081	Weak	0.121	Weak	0.086	Weak	0.039	Weak
	Sichuan	0.092	Weak	0.123	Weak	0.084	Weak	0.018	Weak	0.012	Weak
	Guizhou	0.25	Weak	0.249	Weak	0.126	Weak	0.11	Weak	0.067	Weak
	Yunnan	0.201	Weak	0.124	Weak	−0.088	Weak	−0.035	Weak	0.05	Weak
	Shaanxi	0.565	Weak	0.585	Weak	0.532	Weak	0.426	Weak	0.381	Weak
	Gansu	0.573	Weak	0.405	Weak	0.317	Weak	0.239	Weak	0.223	Weak
	Qinghai	0.355	Weak	0.571	Weak	0.284	Weak	0.352	Weak	0.264	Weak
	Inner Mongolia	0.782	Weak	0.537	Weak	0.512	Weak	0.791	Weak	1.197	Expansive
	Ningxia	1.015	Expansive	0.893	Expansive	0.786	Weak	0.862	Expansive	1.007	Expansive
	Xinjiang	0.597	Weak	0.742	Weak	0.79	Weak	0.757	Weak	0.637	Weak
	Mean	0.498	Weak	0.425	Weak	0.332	Weak	0.347	Weak	0.368	Weak
Whole country	Mean	0.49	Weak	0.339	Weak	0.28	Weak	0.265	Weak	0.293	Weak

Note: Strong represents strong decoupling, weak represents weak decoupling, expansive represents expansive coupling.

**Table 2:** Elasticity of Carbon emission driving factors.

Area	Province	Year									
		2009–2011	2011–2013	2013–2015	2015–2017	2017–2019	2009–2011	2011–2013	2013–2015	2015–2017	2017–2019
		*f_p_*					*f_g_*				
Eastern China	Beijing	0.404	0.278	0.227	0.150	0.101	0.434	0.449	0.454	0.440	0.424
	Tianjin	0.230	0.236	0.225	0.184	0.321	0.722	0.614	0.543	0.517	0.519
	Hebei	0.076	0.071	0.080	0.073	0.083	0.846	0.786	0.732	0.674	0.693
	Shandong	0.056	0.048	0.053	0.059	0.071	0.874	0.801	0.793	0.756	0.786
	Jiangsu	0.059	0.043	0.037	0.031	0.028	0.887	0.830	0.777	0.708	0.656
	Shanghai	0.772	0.553	0.360	0.237	0.162	0.167	0.345	0.447	0.494	0.481
	Zhejiang	0.140	0.098	0.082	0.076	0.078	0.757	0.718	0.680	0.626	0.559
	Fujian	0.066	0.056	0.060	0.055	0.048	0.875	0.766	0.737	0.648	0.603
	Guangdong	0.273	0.186	0.153	0.132	0.119	0.672	0.672	0.640	0.604	0.552
	Hainan	0.032	0.042	0.053	0.049	0.050	0.881	0.740	0.747	0.648	0.605
Northeast China	Liaoning	0.035	0.022	0.017	0.023	0.014	0.856	0.773	0.745	0.845	0.900
	Jilin	0.008	0.006	0.005	−0.008	−0.031	0.926	0.806	0.743	0.725	0.841
	Heilongjiang	0.005	0.004	−0.005	−0.012	−0.036	0.879	0.809	0.803	0.796	0.874
Central China	Shanxi	0.098	0.090	0.104	0.085	0.086	0.777	0.760	0.749	0.735	0.770
	Henan	−0.030	−0.013	−0.001	0.006	0.009	0.956	0.821	0.754	0.653	0.549
	Hubei	0.015	0.016	0.019	0.020	0.016	0.908	0.740	0.668	0.608	0.537
	Hunan	0.063	0.052	0.051	0.047	0.040	0.820	0.711	0.654	0.613	0.545
	Anhui	−0.055	−0.021	0.002	0.014	0.017	0.915	0.828	0.761	0.677	0.570
	Jiangxi	0.027	0.027	0.031	0.032	0.030	0.883	0.818	0.773	0.708	0.646
Western China	Guangxi	−0.109	−0.044	−0.014	0.006	0.017	1.119	0.989	0.861	0.843	0.806
	Chongqing	0.044	0.041	0.041	0.040	0.036	0.857	0.687	0.635	0.558	0.481
	Sichuan	−0.035	−0.012	0.002	0.009	0.010	0.868	0.770	0.699	0.595	0.517
	Guizhou	−0.209	−0.086	−0.048	−0.027	−0.016	1.079	0.853	0.695	0.597	0.506
	Yunnan	0.031	0.029	0.029	0.029	0.024	0.834	0.720	0.592	0.544	0.489
	Shaanxi	−0.017	−0.003	0.006	0.013	0.017	0.934	0.884	0.843	0.765	0.713
	Gansu	−0.064	−0.027	−0.015	−0.003	0.003	0.989	0.867	0.813	0.750	0.697
	Qinghai	0.039	0.048	0.051	0.061	0.061	0.834	0.831	0.709	0.706	0.643
	Inner Mongolia	0.061	0.050	0.052	0.082	0.087	0.902	0.839	0.821	0.873	0.959
	Ningxia	0.050	0.068	0.081	0.089	0.104	0.952	0.903	0.853	0.862	0.898
	Xinjiang	0.048	0.065	0.107	0.122	0.114	0.873	0.863	0.828	0.791	0.732
Area	Province	***f_e_***					***f_s_***				
Eastern China	Beijing	−0.941	−0.860	−0.713	−0.639	−0.537	−0.055	−0.061	−0.072	−0.067	−0.051
	Tianjin	−0.207	−0.388	−0.455	−0.481	−0.387	−0.013	−0.003	−0.017	−0.022	−0.045
	Hebei	−0.411	−0.495	−0.572	−0.591	−0.505	0.002	−0.003	−0.009	−0.008	−0.007
	Shandong	−0.424	−0.528	−0.405	−0.375	−0.314	−0.025	−0.013	−0.017	−0.024	−0.011
	Jiangsu	−0.283	−0.398	−0.428	−0.434	−0.429	0.003	−0.003	−0.006	−0.009	−0.008
	Shanghai	−0.465	−0.484	−0.617	−0.578	−0.546	−0.008	−0.015	−0.024	−0.025	−0.016
	Zhejiang	−0.535	−0.623	−0.608	−0.551	−0.512	−0.014	−0.015	−0.018	−0.016	−0.013
	Fujian	−0.276	−0.495	−0.428	−0.451	−0.388	−0.026	−0.024	−0.013	−0.011	−0.006
	Guangdong	−0.389	−0.566	−0.572	−0.509	−0.484	0.039	0.022	0.009	0.004	0.002
	Hainan	−0.341	−0.532	−0.402	−0.453	−0.419	−0.032	−0.019	−0.002	−0.005	−0.001
Northeast China	Liaoning	−0.508	−0.580	−0.604	−0.525	−0.300	−0.014	−0.012	−0.010	−0.018	−0.014
	Jilin	−0.327	−0.546	−0.616	−0.615	−0.675	0.002	−0.001	−0.003	−0.007	−0.009
	Heilongjiang	−0.539	−0.619	−0.603	−0.585	−0.614	−0.007	0.005	−0.002	0.002	0.001
Central China	Shanxi	−0.525	−0.473	−0.453	−0.391	−0.278	−0.001	−0.006	−0.009	−0.008	−0.005
	Henan	−0.419	−0.633	−0.615	−0.605	−0.573	−0.005	−0.019	−0.011	−0.014	−0.016
	Hubei	−0.364	−0.613	−0.575	−0.524	−0.456	0.028	0.000	−0.005	−0.005	−0.007
	Hunan	−0.502	−0.600	−0.560	−0.511	−0.495	−0.011	−0.012	−0.015	−0.014	−0.015
	Anhui	−0.581	−0.482	−0.470	−0.453	−0.411	−0.014	−0.014	−0.014	−0.014	−0.011
	Jiangxi	−0.370	−0.394	−0.391	−0.399	−0.382	−0.015	−0.022	−0.019	−0.017	−0.015
Western China	Guangxi	0.038	−0.171	−0.330	−0.286	−0.282	0.009	0.016	0.000	0.000	0.003
	Chongqing	−0.408	−0.619	−0.531	−0.491	−0.456	−0.011	−0.028	−0.024	−0.020	−0.021
	Sichuan	−0.722	−0.621	−0.597	−0.563	−0.494	−0.020	−0.014	−0.020	−0.023	−0.021
	Guizhou	−0.616	−0.513	−0.511	−0.451	−0.412	−0.004	−0.005	−0.009	−0.009	−0.011
	Yunnan	−0.643	−0.617	−0.690	−0.593	−0.452	−0.020	−0.008	−0.018	−0.015	−0.010
	Shaanxi	−0.358	−0.308	−0.326	−0.356	−0.351	0.006	0.011	0.008	0.004	0.002
	Gansu	−0.351	−0.423	−0.467	−0.493	−0.467	0.000	−0.013	−0.013	−0.015	−0.011
	Qinghai	−0.502	−0.303	−0.460	−0.394	−0.418	−0.016	−0.004	−0.015	−0.020	−0.022
	Inner Mongolia	−0.184	−0.362	−0.372	−0.175	0.135	0.004	0.010	0.010	0.012	0.016
	Ningxia	0.010	−0.083	−0.151	−0.092	0.001	0.003	0.004	0.003	0.003	0.004
	Xinjiang	−0.332	−0.188	−0.142	−0.160	−0.214	0.007	0.002	−0.003	0.004	0.005

## Data Availability

Data available in a publicly accessible repository that does not issue DOIs. Publicly available datasets were analyzed in this study. This data can be found here: [https://navi.cnki.net/knavi/yearbooks/YINFN/detail, accessed on 4 January 2022] [https://navi.cnki.net/knavi/yearbooks/YCXME/detail, accessed on 4 January 2022].
